# Modeling of prolactin response following dopamine D_2_ receptor antagonists in rats: can it be translated to clinical dosing?

**DOI:** 10.1002/prp2.364

**Published:** 2017-11-21

**Authors:** Amit Taneja, An Vermeulen, Dymphy R. H. Huntjens, Meindert Danhof, Elizabeth C. M. De Lange, Johannes H. Proost

**Affiliations:** ^1^ Division of Pharmacokinetics, Toxicology and Targeting Groningen Research Institute of Pharmacy University of Groningen Antonius Deusinglaan 1 9713 AV Groningen The Netherlands; ^2^ Division of Janssen Pharmaceutica NV Clinical Pharmacology and Pharmacometrics Janssen Research and Development Beerse Belgium; ^3^ Department of Pharmacology Leiden Academic Center for Drug Research Leiden University Leiden The Netherlands

**Keywords:** Agonist–antagonist interaction model, dopamine D2 antagonists, precursor pool model, prolactin, receptor occupancy, translational modeling

## Abstract

Prolactin release is a side effect of antipsychotic therapy with dopamine antagonists, observed in rats as well as humans. We examined whether two semimechanistic models could describe prolactin response in rats and subsequently be translated to predict pituitary dopamine D_2_ receptor occupancy and plasma prolactin concentrations in humans following administration of paliperidone or remoxipride. Data on male Wistar rats receiving single or multiple doses of risperidone, paliperidone, or remoxipride was described by two semimechanistic models, the precursor pool model and the agonist–antagonist interaction model. Using interspecies scaling approaches, human D_2_ receptor occupancy and plasma prolactin concentrations were predicted for a range of clinical paliperidone and remoxipride doses. The predictions were compared with corresponding observations described in literature as well as with predictions from published models developed on human data. The pool model could predict D_2_ receptor occupancy and prolactin response in humans following single doses of paliperidone and remoxipride. Tolerance of prolactin release was predicted following multiple doses. The interaction model underpredicted both D_2_ receptor occupancy and prolactin response. Prolactin elevation may be deployed as a suitable biomarker for interspecies translation and can inform the clinical safe and effective dose range of antipsychotic drugs. While the pool model was more predictive than the interaction model, it overpredicted tolerance on multiple dosing. Shortcomings of the translations reflect the need for better mechanistic models.

**Abbreviations**
AAI: agonist–antagonist interaction model
D_2_: dopamine‐2 receptor
ECu_50_: effective unbound concentration at half‐maximal effect
KI: inhibition constant
PA: paliperidone
PD: pharmacodynamics
PK: pharmacokinetics
PP: precursor pool model
REM: remoxipride
RI: risperidone
RO: receptor occupancy, in %

## Introduction

Antipsychotics are the standard of care for schizophrenia, and bring about their effects at least in part by binding to central D_2_ receptors. Aside from central D_2_ receptor antagonism, these drugs also bind to peripheral D_2_ receptors located in the pituitary lactotrophs, which in turn leads to plasma prolactin elevation (Peuskens et al. 2014). This phenomenon is similar across species and thus prolactin elevation may be deployed as a suitable biomarker for interspecies translation (Ben‐Jonathan et al. 2008).

While interspecies scaling of pharmacokinetic (PK) parameters is common in drug development, limited information is available on prediction of pharmacodynamic (PD) parameters (Boxenbaum 1982; Lepist and Jusko 2004). Zuideveld et al. (2007) were able to predict the hypothermic and corticosterone releasing effects of flesinoxan and buspirone in humans using a mechanistic PKPD model developed on rat data. Yet, such predictions are not always possible, due to interspecies differences. Yassen et al. (2007a,b) were able to translate the respiratory depressant effects from rats to humans for buprenorphine, the same was not the case for the antinociceptive effects. This is due to different opioid mu receptor involved in the antinociceptive activity and respiratory depressant effects. More recently, the PD of prolactin following remoxipride administration was successfully extrapolated from rats to humans (Stevens et al. 2012).

We fitted two mechanism‐based models, the precursor pool (PP) model and the agonist– antagonist interaction (AAI) model, to describe prolactin response in rats following single doses of risperidone (RI) or paliperidone (PA), or two doses of remoxipride (REM) (Taneja et al. 2016a,b). While the AAI model predicted prolactin response following multiple doses in rats better than the PP model, the latter described the time course of receptor occupancy better. To the best of our knowledge, the interspecies scaling of prolactin response has been described only for remoxipride (Stevens et al. 2012), whereas rat to human scaling has not been published for the AAI model. Given this background, the aim of this work was to evaluate the predictive performance of either model applying standard systems pharmacology interspecies scaling approaches (Mager et al. 2009; Stevens et al. 2012; Petersson et al. 2013). We further examine the translatability of these PKPD models to predict pituitary dopamine receptor occupancy (D_2_ RO) and plasma prolactin response at steady‐state concentrations of PA and REM. The translational value of either model in predicting efficacy and safety in humans is compared with published reports. The translational value of prolactin is examined as well as its linkage to receptor occupancy. The overarching aim of this work was to explore whether interspecies translation of prolactin response could inform dosing for subsequent first‐in‐human studies.

## Materials and Methods

The experimental methods, as well as preclinical model fitting have been presented elsewhere (Taneja et al. 2016a,b). Here, we describe the models that were fitted and the translational strategy for scaling the fitted models from rats to humans.

### Pharmacodynamic models

The PP model (Fig. S1) is an indirect response model comprising of two hypothetical PD compartments, represented by two differential equations describing the turnover of prolactin in the lactotroph pool and in plasma, respectively. The PP model hypothesizes tolerance development following repeated doses of D_2_ antagonists to be a result of depletion of the lactotroph pool (Movin‐Osswald and Hammarlund‐Udenaes 1995). The turnover of prolactin in the pool and in plasma is described as follows:

(1) $$\frac{dC_{\mathrm{pool}}}{dt}=R_{\mathrm{form}}\cdot \left(1+\text{PF}\right)-K_{\mathrm{base}}\cdot \left(1+\text{DE}\right)\cdot C_{\mathrm{pool}}$$

(2) $$\frac{dC_{\mathrm{prl}}}{dt}=K_{\mathrm{base}}\cdot \left(1+\text{DE}\right)\cdot C_{\mathrm{pool}}-K_{\mathrm{out}}\cdot C_{\mathrm{prl}}$$

where *C*_pool_ and *C*_prl_ are the concentration of prolactin in the lactotroph pool and plasma, respectively, *R*_form_ is the zero‐order rate constant for prolactin synthesis, *K*_base_ is the first‐order rate constant of prolactin release from the pool, and *K*_out_ is the first‐order rate constant of elimination of prolactin from plasma.

Dopamine antagonists cause release of prolactin from the pool, parameterized as drug effect DE given by the following function:

(3) $$\text{DE}=E_{\max}\cdot \frac{{\text{Cu}}^{\gamma }}{{\text{ECu}}_{50}^{\gamma }+{\text{Cu}}^{\gamma }}$$

where *E*_max_ is the maximum increase in the prolactin release from the pool, ECu_50_ is the unbound drug concentration at half‐maximal effect, Cu is the unbound concentration of the drug in plasma (unless otherwise mentioned), and γ is a slope factor. Once the final PP model was developed, this model was modified to relate drug effect to pituitary receptor occupancy (RO), rather than to unbound drug concentration. Drug effect can be represented in terms of RO as per the following expression:

(4) $$\text{DE}=E_{\max}\cdot \frac{{\left(\frac{\mathrm{RO}}{100-\mathrm{RO}}\right)}^{\gamma }}{{\left(\frac{{\mathrm{RO}}_{50}}{100-{\mathrm{RO}}_{50}}\right)}^{\gamma }+{\left(\frac{\mathrm{RO}}{100-\mathrm{RO}}\right)}^{\gamma }}$$

RO_50_ is defined as RO for Cu* *= ECu_50_ (additional details can be found in the supplemental section).

The second published model is the AAI model (Fig. S2) which has been used to describe clinical data following administration of D_2_ antagonists to patients and human healthy volunteers (Friberg et al. 2009b; Ma et al. 2010). This model describes the competition between the concentrations of hypothetical (unobserved) dopamine (DA) and the dopamine antagonist at the D_2_ receptor. Prolactin stimulates the production of DA while the hypothetical DA concentration inhibits prolactin release. This model was originally proposed by Bagli and colleagues (Bagli et al. 1999) and subsequently modified (Friberg et al. 2009a) to additionally model the diurnal variation in prolactin release.

The turnover of prolactin in plasma is described by:

(5) $$\frac{dC_{\mathrm{prl}}}{dt}=K_{\mathrm{in},0}\cdot \left(\left(1+{\text{DAs}}_{0}\right)\cdot \left(1-\frac{\text{DAs}}{\text{DAs}+\frac{\text{Cu}}{\text{KI}}+1}\right)+f\left(\text{DIU}\right)\right)-K_{\mathrm{out}}\cdot C_{\mathrm{prl}}$$

where *K*_in,0_ is the basal prolactin release rate, DAs_0_ and DAs are the hypothetical scaled dopamine concentrations at baseline and at time *t*, respectively, KI is the drug potency parameter, and *f*(DIU) is a double cosine function to describe the diurnal variation in prolactin release (Friberg et al. 2009b).

The time course of hypothetical dopamine (DAs) is parameterized as follows:

(6) $$\frac{\text{dDAs}}{dt}=K_{\mathrm{DA}}\cdot {\text{DAs}}_{0}\cdot {\left(\frac{C_{\mathrm{prl}}}{C_{\mathrm{prl},0}}\right)}^{\gamma }-K_{\mathrm{DA}}\cdot \mathrm{DAs}$$

*K*_DA_ is the first‐order rate turnover constant for hypothetical dopamine and the ratio *C*_prl_/*C*_prl,0_ is a positive feedback factor of prolactin on dopamine secretion, and *γ* the slope parameter of the positive feedback. In published studies to date, the DAs_0_ parameter was fixed to 10,000, as it could not be estimated (Friberg et al. 2009b). We evaluated if the data was informative enough to be able to estimate this parameter.

According to the theory of competitive receptor interaction, the receptor occupancy of the D_2_ antagonist drug can be derived from the following expression:

(7) $$\mathrm{RO}=\frac{\frac{\mathrm{Cu}}{\mathrm{KI}}}{\mathrm{DAs}+\frac{\mathrm{Cu}}{\mathrm{KI}}+1}\cdot 100$$

and the receptor occupancy of dopamine is described by

(8) $${\text{RO}}_{\mathrm{dopamine}}=\frac{\text{DAs}}{\text{DAs}+\frac{\mathrm{Cu}}{\mathrm{KI}}+1}\cdot 100$$

A step‐by‐step derivation of equations (7) and (8) can be found in the accompanying supplemental material.

In vitro experimental KI values for all three compounds in both rat and human species were available to us (Taneja et al. 2016b). We estimated RO_50_ using both the available experimental rat KI values as well as estimated values obtained by fitting the AAI model to the available data (Taneja et al. 2016a).

### Rat‐to‐human translations

Predicted human unbound population plasma concentrations were used as the driving force for the receptor occupancy and prolactin response, and these were based on human PK models for PA (OROS PA formulation) and REM, previously described in the literature (Samtani et al. 2011; Johnson 2012; Stevens et al. 2012). In case of REM, the PK followed two‐compartment first‐order kinetics, with the drug being administered intravenously. The PK of the OROS PA formulation has been described by a one‐compartment model with sequential zero‐ and first‐order absorption (Johnson 2012).

For both models, the system‐specific rate constants (*R*_form_, *K*_base_, and *K*_out_ in the PP model; *K*_in,0_, *K*_out_, and *K*_DA_ in the AAI model) were scaled allometrically, as per the following expression.

(9) $$\frac{K_{\mathrm{hum}}}{K_{\mathrm{rat}}}={\left(\frac{{\text{BW}}_{\mathrm{hum}}}{{\text{BW}}_{\mathrm{rat}}}\right)}^{b}$$

where *K*_hum_ and *K*_rat_ refer to turnover constants in humans and rats, respectively. BW_hum_ and BW_rat_ are the respective body weights taken to be 70 kg and 0.28 kg. *b* is the allometric exponent fixed to −0.25 (Lepist and Jusko 2004; Anderson and Holford 2008).

Not all model parameters were scaled as described above and for these, alternative strategies were applied as explained hereunder, separately for each model.

#### PP model

The *E*_max_ was fixed for each compound to the value estimated by Movin‐Osswald and Hammarlund‐Udenaes (1995).

There are similarities between the neuroendocrine control of prolactin release between rats and humans, and much of what is known about the underlying physiology is based on studies in rodent models (Ben‐Jonathan et al. 2008). Given this fact, the system‐specific parameter RO_50_ was assumed to remain constant across compounds and species and was fixed to the value obtained from the fits to rat data.

#### AAI model

Two approaches were investigated to obtain the human KIs. Petersson and colleagues have shown that human in vitro experimental KI values for antipsychotics correlate well with corresponding values estimated in vivo (*r* ^2^ = 0.94, *P* < 0.001) for five different antipsychotics, using data from 16 clinical trials (Petersson et al. 2013). In the first approach, these values were fixed to those from in vitro experimental human values (Taneja et al. 2016b).

The second approach was based on integrating estimated and experimental information given by the following function (Johnson 2012; Johnson et al. 2016):

(10) $${\text{KI}}_{\mathrm{hum}}=\text{in vitro}\;{\text{KI}}_{\mathrm{hum}}\cdot \frac{{\text{KI}}_{\mathrm{rat}}}{\text{in vitro}\;{\text{KI}}_{\mathrm{rat}}}$$

where KI_hum_ is the scaled human potency, in vitro KI_hum_ and in vitro KI_rat_ are the experimental KI values for human and rat, respectively, and KI_rat_ is the estimated potency parameter from rat data fits.

As DAs_0_ is a scaled concentration, no scaling was attempted. Rather, the estimated value from rat data (10.9) and the published estimated human value (10 000) were both tested.

RO and prolactin profiles were predicted for both models using the functions described earlier. For PA, RO and prolactin responses for the following doses were predicted: 1.5, 3, 4.5, 6, 9, and 12 mg given once daily for 8 days. For REM, the chosen dose range was 50, 100, 150, 300, 450, and 600 mg once daily for 8 days. The chosen dose ranges are based on the clinical therapeutic range (Kane 1993). Farde and colleagues investigated an alternative dose regimen of 100 mg thrice daily and 200 mg twice daily for REM in a PET study on healthy human volunteers (Farde et al. 1988).

Using the model that best described RO and prolactin response, we additionally predicted pharmacodynamic responses with this alternative regimen over a period of 8 days. Rapid adaptation is a feature of the PP model and hence, as an additional scenario for PA, we investigated the effect of increasing the dosing interval to 7 days between two consecutive doses (Mager and Jusko 2007).

### Comparison of the predictions with published human models

Model predictions were compared with data gleaned from published literature. The benchmark data and the rationale for selection are described hereunder.

De Ridder (2005) developed a population PK model using data from a four‐way crossover trial in 32 healthy subjects comparing single doses of an experimental controlled‐release formulation with an oral solution. Using this model, a virtual population of 2000 patients was simulated and peak as well as average D_2_ RO were predicted. We compared RO predictions from the translational PP model with those of De Ridder. In vitro values for D_2_ RO from literature (Johnson 2012; Johnson et al. 2016) were overlaid on the predicted time course of RO we reported.

Average population plasma time course profiles of prolactin were simulated for the original PP and AAI models using the parameter estimates reported in literature (Movin‐Osswald and Hammarlund‐Udenaes 1995; Friberg et al. 2009b; Ma et al. 2010). These models were fitted to human data and hence considered as benchmark models. The Friberg model has a function for the diurnal rhythm, which was identifiable in humans. In the current comparison, predictions with this model are done without the diurnal rhythm function. The model parameters used for these simulations are presented in Table 1.

**Table 1:** Translated parameter estimates using the PP and AAI model as compared to published findings

	Movin‐Osswald and Hammarlund‐Udenaes (1995)	Friberg et al. (2009b) and Ma et al. (2010)	Our findings: translation from rat to human
PP model			
*R*_form_ (ng·mL^−1^·h^−1^)	16	26.5	12.4^a^
*K*_base_ (h^−1^)	0.105	0.11	0.060^a^
*K*_out_ (h^−1^)	1.3	2.09	1.67^a^
*E*_max_	66^b^	NE	66^c^
Slope (L·mg^−1^)	NE	4.08	NA
RO_50_ (%)	NA	NA	56.3^d^
*C*_pool,0_ (ng·mL^−1^)	144^e^	246	207^f^
*C*_prl,0_ (ng·mL^−1^)	9.4^e^	12.7	7.42^g^
EC50_PA (*μ*mol/L)	0.276^h^	NE	NA
EC50_REM (*μ*mol/L)	22	NE	NA

	Friberg et al. (2009b) and Ma et al (2010)	Friberg et al. (2009b) and Ma et al. (2010)	Our findings: translation from rat to human
AAI model			
*K*_in,0_ (ng·mL^−1^·h^−1^)	5.09^i^	7.33^i^	7.59^a^
*K*_out_ (h^−1^)	0.664	0.803	1.45^a^
*K*_DA_ (h^−1^)	0.156	0.134	0.99^a^
DAs_0_	10,000	10,000	10.9^d^
*γ*	1.44	2.31	1^j^
*C*_prl,0_ (ng·mL^−1^)	7.67	9.13	5.23^i^
KI_PA (nmol/L) (unbound)	1.04^k^	NE	8.43^l^
KI_REM (nmol/L) (unbound)	NE	37.0^m^	50.5^n^

NE, not estimated; NA, not applicable.

a Calculated by allometric scaling (eq. (9)) using values in the rat Taneja et al. (2016a), with BW_hum_ = 70 kg, BW_rat_ = 0.28 kg, and *b* = −0.25 Lepist and Jusko (2004).

b Value reported for *E*_max_ model.

c Fixed for each compound to the value estimated in humans Movin‐Osswald and Hammarlund‐Udenaes (1995).

d Fixed for each compound to the value estimated in rats Taneja et al. (2016a).

e In the original pool model, mass balance was not taken into account.

f Calculated from *C*_pool,0_ =* R*_form_/*K*_base_.

g Calculated from *C*_prl,0_ =* R*_form_/*K*_out_.

h See methods for scaling of EC50 (eq. (11)): in vitro KI_PA,hum_ **= 2.08 nmol/L Taneja et al. (2016b), in vitro KI_rem,hum_ **= 165.75 nmol/L Taneja et al. (2016b).

i Calculated from *C*_prl,0_ = *K*_in,0_/*K*_out_.

j Fixed to 1 since slope factor *γ* could not be estimated in rats (Taneja et al. 2016a).

k Calculated from KI = 1.96 ng·mL^−1^ Friberg et al. (2009b) and Ma et al. (2010) and protein binding 77.4% Taneja et al. (2016b) (molecular weight PA = 426.48).

l See methods for scaling of KI (eq. (10)) PA: in vitro KI_rat_ *= *2.74 nmol/L Taneja et al. (2016b), in vitro KI_hum_ = 2.08 nmol/L Taneja et al. (2016b), in vivo (rat) KI = 11.1 nmol/L Taneja et al. (2016a).

m Calculated from KI = 0.0687 mg·L^−1^ Friberg et al. (2009b) and Ma et al. (2010) and protein binding 80% Taneja et al. (2016b) (molecular weight REM = 371.26).

n See methods for scaling of KI (eq. (10)) REM: in vitro KI_rat_ *= *370.66 nmol/L Taneja et al. (2016b), in vitro KI_hum_ = 165.75 nmol/L Taneja et al. (2016b)), in vivo (rat) KI = 113 nmol/L Taneja et al. (2016a).

To the best of our knowledge, fitting of the PP model to clinical PA data has not been published. In the current analysis, we used the following expression to derive the putative human EC_50_ of PA:

(11) $${\text{EC}}_{50,\mathrm{PA},\mathrm{hum}}={\text{EC}}_{50,\mathrm{REM},\mathrm{hum}}\cdot \frac{\text{in vitro}\;{\text{KI}}_{\mathrm{PA},\mathrm{hum}}}{\text{in vitro}\;{\text{KI}}_{\mathrm{REM},\mathrm{hum}}}$$

where EC_50,PA,hum_ and EC_50,REM,hum_ are the human EC_50s_ for PA and REM, respectively, and in vitro KI_PA,hum_ and in vitro KI_REM,hum_ are the corresponding human *KIs*.

Berwaerts et al. (2010) compared the prolactin releasing potential of an ER preparation of paliperidone with that of an IR formulation of risperidone. Given that we used PK parameters from a similar formulation for our simulations, we compared our model‐predicted prolactin response with that observed by Berwaerts and colleagues. The mean observed plasma prolactin profiles for the 12 mg OROS PA formulation from a published source were extracted using WebplotDigitizer, and overlaid on the predicted prolactin profiles for both models (Rohatagi 2014).

### Software

Simulation was performed with NONMEM version 7.2.0 (Icon Development solutions, Hanover, MD, USA (Beal et al. 2009)) in conjunction with PsN version 3.7.6 which was used as a NONMEM interface (Lindbom et al. 2004). R version 3.02 along with package Xpose 4 was used for data manipulation, and statistical and graphical summaries (Lindbom et al. 2004; R Core Team, 2015). Microsoft Excel 2007 was used for the simulations in the validation exercise. WebplotDigitizer was used to extract published data (Rohatagi 2014). Additional details on the methods such as the experimental procedure and bioanalysis, model parameterization and model building can be found in the published literature (Taneja et al. 2016a).

## Results

### PP model predictions for PA

**Figure 1:**
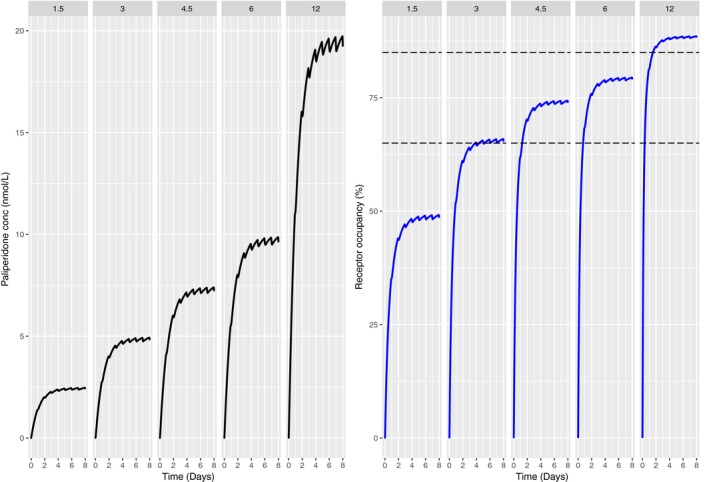
Predicted human plasma concentration profiles (left panel) and RO profiles (right panel) with the translational PP model following once daily dosing of PA 1.5, 3, 4.5, 6, 12 mg/day for 8 days. Dashed lines (right panel) show the zone of observed experimental in vitro RO (Johnson 2012).

The predicted plasma PA concentration time course and the corresponding RO time course for the OROS formulation are depicted in Figure 1. PA OROS has a zero‐order release of more than 20 h and the half‐life of PA is ~28 h (Johnson 2012; Rodriguez‐Martinez and Quilo 2013). Steady state is thus reached at around 4 days after dosing. There is little fluctuation between the minimum and maximum plasma concentrations. The same phenomenon is predicted for the RO as well. The predicted RO is in agreement with clinically observed central RO following daily doses of 9 mg (Johnson 2012).

**Figure 2:**
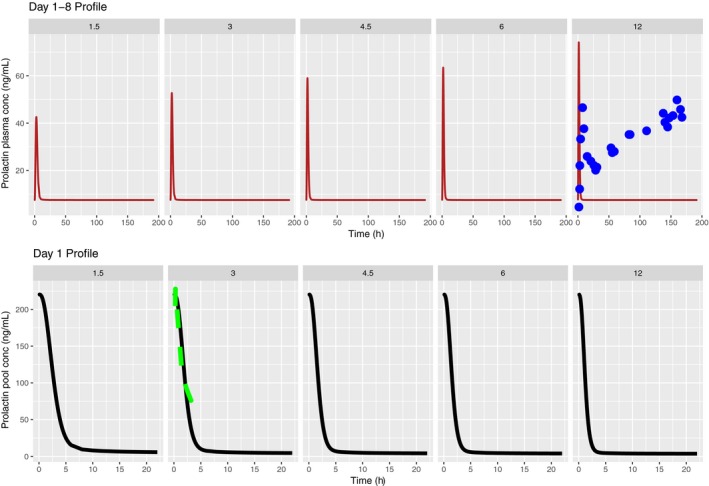
Predicted human plasma prolactin profiles (upper panel) and lactotroph prolactin profiles (lower panel) with the translational PP model following once daily dosing of PA 1.5, 3, 4.5, 6, 12 mg/day for 8 days. Blue dots in the right panel show the mean observed human plasma prolactin profiles over 7 days (Berwaerts et al. 2010). Green dashed line in the lower panel is the predicted human lactotroph prolactin profiles (Movin‐Osswald and Hammarlund‐Udenaes 1995).

**Figure 3:**
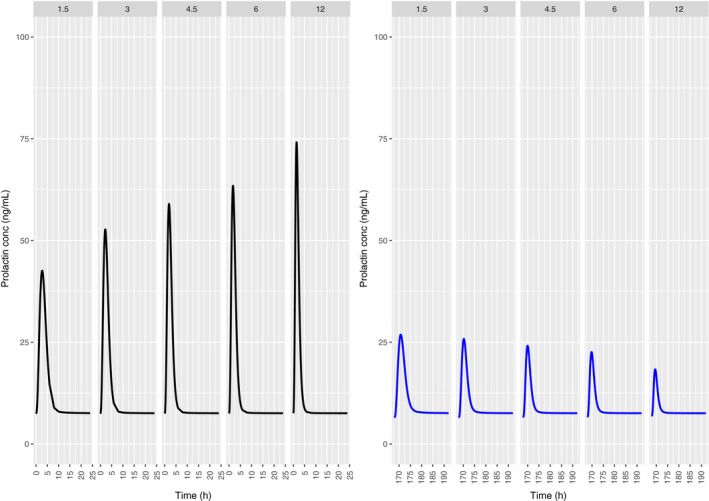
Effect of increase in dose interval on the predicted human plasma prolactin profiles with the translational PP model. Doses of PA 1.5, 3, 4.5, 6, 12 mg/day are administered on day 1 (left panel) and day 8 (right panel).

Figure 2 upper panels show the predicted prolactin plasma time course over 8 days for the PP model as well as the corresponding lactotroph prolactin time course. Mean observations from a multiple dose study in healthy volunteers receiving PA OROS 12 mg daily for 7 days were extracted by digitization and are overlaid on the plasma prolactin time course predictions (Berwaerts et al. 2010). In the lower panels, the prolactin lactotroph time course predictions based on a healthy volunteer dataset are overlaid on our predictions, showing good agreement between both models on day 1 (Movin‐Osswald and Hammarlund‐Udenaes 1995). Tolerance following multiple doses is predicted, as evident from the flat prolactin concentration in Figure 2. However, by increasing the dosing interval between successive doses to 7 days, diminished tolerance is predicted, with the appearance of a smaller peak on day 8 (Fig. 3).

### PP model predictions for REM

**Figure 4:**
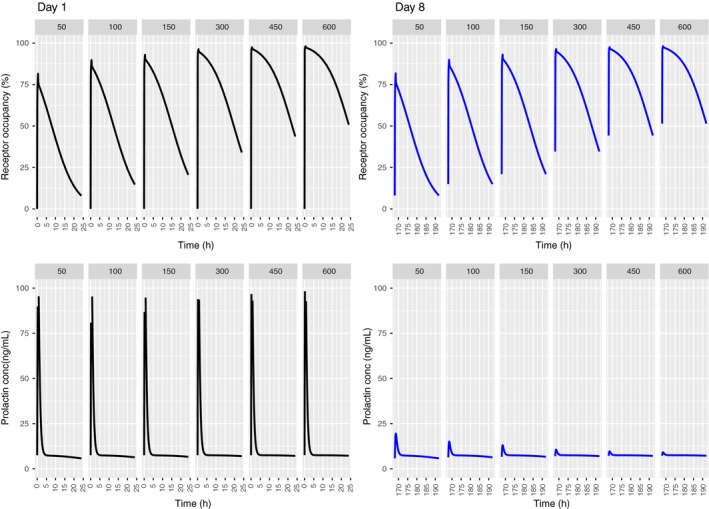
Predicted human RO profiles (upper panels) and plasma prolactin profiles (lower panels) with the translational PP model following once daily dosing of REM 50, 100, 150, 300, 450, 600 mg/day for 8 days. Day 1 (left panels) and day 8 (right panels) profiles are depicted.

The time course of the RO and corresponding prolactin release are shown in Figure 4. RO is predicted to increase nonlinearly. In contrast to PA, RO decreases during the dosing interval, although not completely returning to baseline. With increasing doses, the prolactin concentrations are also predicted to increase nonlinearly. As in the case of PA, tolerance of prolactin response is predicted after the first dose, but to a lesser extent than for PA. Partial recovery of the pool is predicted for doses up to 300 mg, although subsequent prolactin peak concentrations are about 10% of those on day 1.

With altered dosing paradigms of 100 mg thrice daily and 200 mg twice daily, respectively, peak RO was 90% and 95%, with median levels being 83% (74–89%) and 89% (72–93%), respectively (Fig. S3). Figures in brackets indicate the 5% and 95% range. With these dosing regimens, complete pool depletion was predicted following the first day of dosing.

For either drug, predictions were not sensitive to RO_50_ values of 56.2% or 28.7%, which are obtained when estimated or in vitro values of KI were used, respectively. Scaling the KI, as per equation (10), resulted in peak plasma prolactin concentrations being 6% higher for REM, while for PA, these were 30% lower.

### AAI model predictions for PA

**Figure 5:**
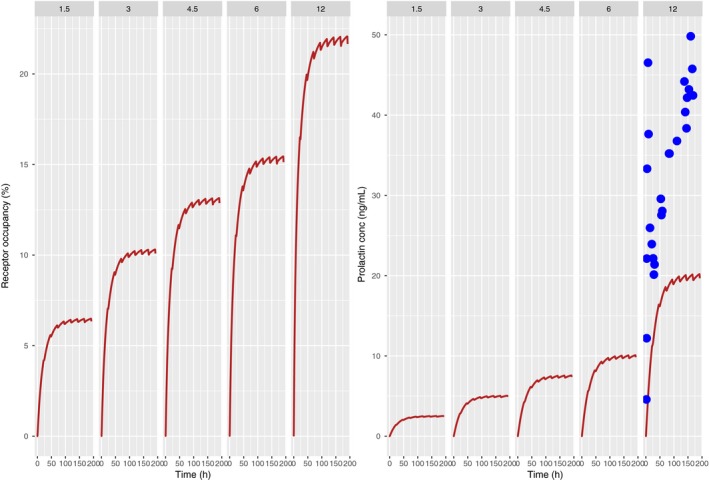
Predicted human RO profiles (left panel) and plasma prolactin profiles (right panel) with the translational AAI model following once daily dosing of PA 1.5, 3, 4.5, 6, 12 mg/day for 8 days. Blue dots in the right panel show the mean observed human plasma prolactin profiles over 7 days (Berwaerts et al. 2010).

Figure 5 shows the predicted RO and prolactin concentrations with the mean observations overlaid. With a DAs_0_ of 10.9 (estimated from the rat), predicted RO was between 7% and 20% across the dose range. No tolerance is evident. It can be seen that the plasma prolactin levels are underpredicted, while tolerance is negligible.

### AAI model predictions for REM

**Figure 6:**
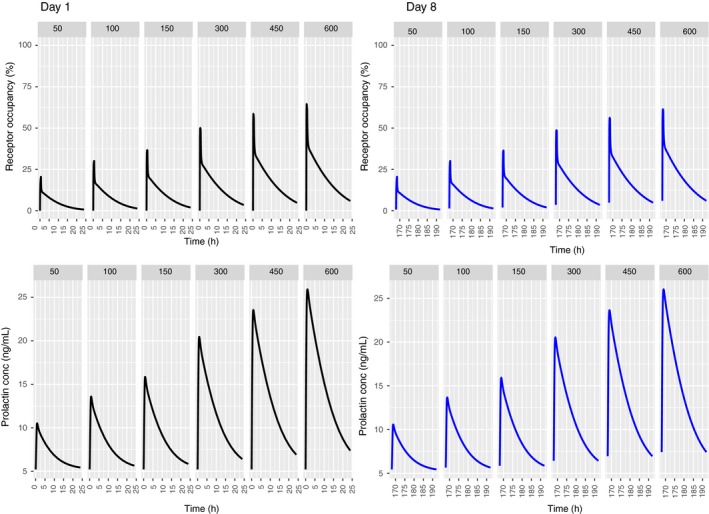
Predicted human RO profiles (upper panels) and plasma prolactin profiles (lower panels) with the translational AAI model following once daily dosing of REM 50, 100, 150, 300, 450, 600 mg/day for 8 days. Day 1 (left panels) and day 8 (right panels) profiles are depicted.

With a DAs_0_ of 10.9, predicted RO ranged between 20% and 60%, the maximum being a little over 60% at the highest dose of 600 mg daily (Fig. 6). The RO is lower on day 8 as compared to day 1, for doses >300 mg. This is due to the increased DAs which is found to be higher at the time of dosing at day 8, when compared to day 1 (DAs_0_). Predicted prolactin concentrations are correspondingly about eightfold lower than those reported by Movin‐Osswald and Hammarlund‐Udenaes (1995) and tolerance is predicted as well at doses >300 mg (Fig. 6).

Fixing the DAs_0_ parameter to the fitted parameter value 10.9 or to 10 000 as proposed by Friberg et al. (2009b) affected the prediction of the RO of the antipsychotic drugs about 1000‐fold, with extremely low levels of RO for a parameter value of 10 000. The predicted prolactin concentrations were not sensitive to these wide variations in this parameter value given that other parameters did not change (data not shown). Scaled KIs increased the *C*_max_ of the predicted plasma prolactin by 20–30%.

### Comparison of the predictions with published literature

Table 1 shows the parameter estimates by our interspecies scaling approach as compared to estimates by fitting of human data as published in the literature. Scaled parameters for both models were in the same ballpark, except for *K*_DA_, which differed by almost one log order.

**Figure 7:**
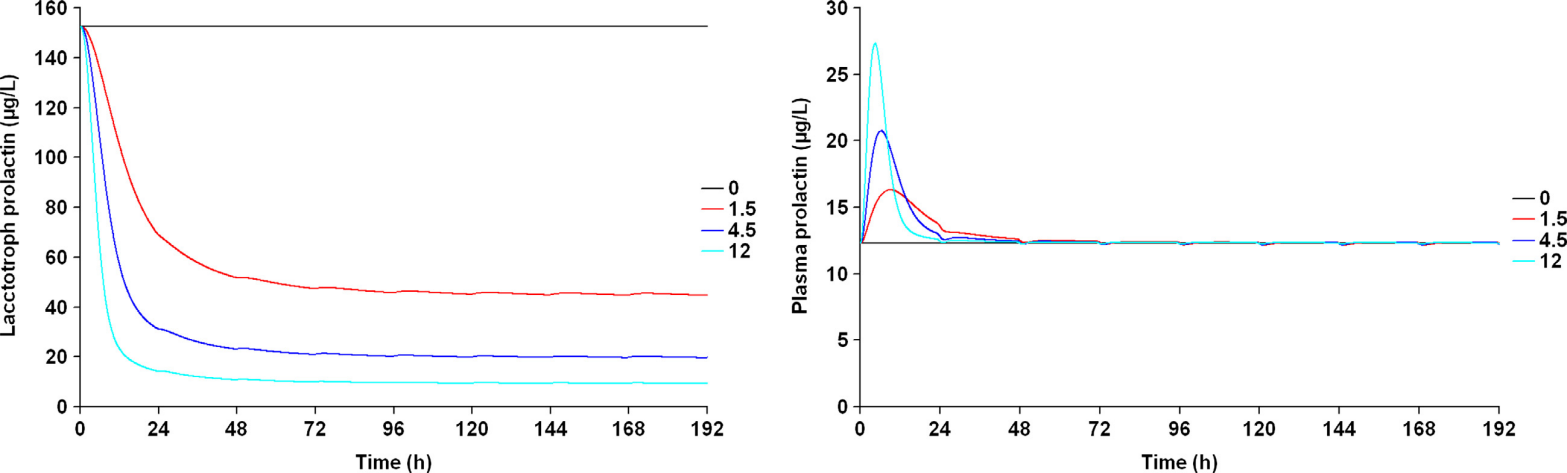
Predicted human lactotroph prolactin profiles (left panel) and plasma prolactin profiles (right panel) with the original PP model (Movin‐Osswald and Hammarlund‐Udenaes 1995) following once daily dosing of PA 0, 1.5, 4.5, 12 mg/day for 8 days.

**Figure 8:**
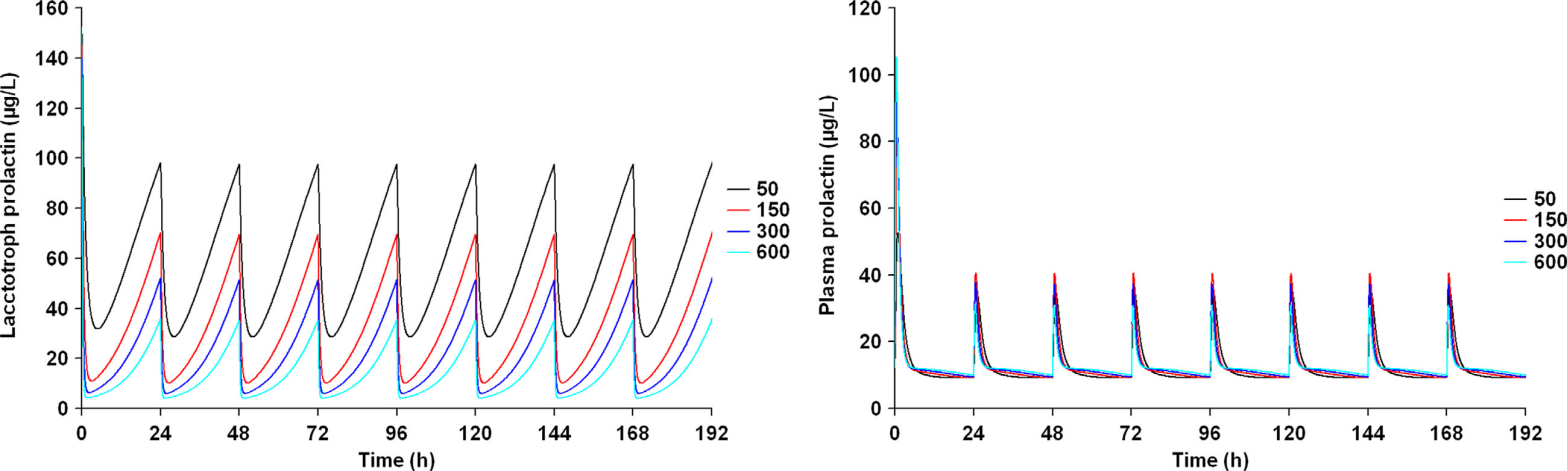
Predicted human lactotroph prolactin profiles (left panel) and plasma prolactin profiles (right panel) with the original PP model (Movin‐Osswald and Hammarlund‐Udenaes 1995) following once daily dosing of REM 50, 150, 300, 600 mg/day for 8 days.

Figure 7 depicts the time course of the predicted pool and plasma prolactin profiles using the original PP model for PA (Movin‐Osswald and Hammarlund‐Udenaes 1995). These predictions are in agreement with those using the interspecies scaling approach (Fig. 2), in that pool depletion is predicted following the first dose of PA. The peak concentrations are lower as compared to Figure 2. Both models predict tolerance following the first dose of PA, although there are differences in its extent. These differences are attributable to different values of the potency parameter used in these simulations. In case of the interspecies scaling approach (Fig. 2), the KI is 2.08 nmol/L, while for simulations with the Movin‐Osswald model, the EC_50_ was 0.276 *μ*mol/L (eq. (11)). In Figure 8 the time course of prolactin in the lactotroph and plasma following 8 daily oral doses of REM is depicted, again using the original model (Movin‐Osswald and Hammarlund‐Udenaes 1995). As compared to the interspecies scaling approach, plasma prolactin profiles on day 1 differ by up to twofold (Fig. 4). On day 1 these are higher with the translational PP model, and lower on day 8, indicating that the translational PP approach predicts tolerance to a greater extent compared to the Movin‐Osswald model. However, predictions with the Movin‐Osswald model also suggest that tolerance is predicted to a lesser extent for REM (Fig. 8) as compared to PA (Fig. 7). The prolactin predictions fluctuate as per the plasma concentration levels for REM, while for PA, prolactin concentrations remain at baseline level after the first dose.

**Figure 9:**
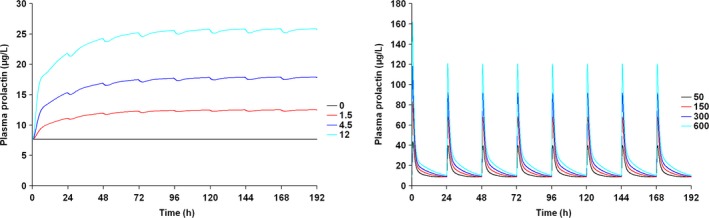
Predicted human plasma prolactin profiles with the original AAI model following once daily dosing of PA 0, 1.5, 4.5, 12 mg/day for 8 days (left panel) (Friberg et al. 2009b) or following once daily dose of REM 50, 150, 300, 600 mg/day for 8 days (right panel) (Ma et al. 2010).

In Figure 9 (left panel) the time course of plasma prolactin following PA administration is depicted using the Friberg et al. (2009a) model without a diurnal rhythm function. Prolactin levels with this model are predicted to be 30% higher than those predicted by the translational AAI model (Fig. 5). The plasma prolactin profile for REM predicted with the Ma AAI model is depicted in the right panel of Figure 9 (Ma et al. 2010). These predictions are similar to those with the Movin‐Osswald model (Fig. 8) (Movin‐Osswald and Hammarlund‐Udenaes 1995). This is logical since both models were fitted by Ma et al. (2010) to the same data. The corresponding translational AAI model underpredicts the prolactin concentrations by almost fivefold, and no tolerance is predicted (Fig. 6). The model parameters used for these comparisons are presented in Table 1 (PD) and Table S1 (PK).

## Discussion

The PP model and the AAI model were comparable in describing the rat data (Taneja et al. 2016a). In order to investigate the translatability of these models, we first investigated the best strategy to scale the model parameters. We applied allometric approaches to scale system‐specific rate constants (*R*_form_, *K*_base_, and *K*_out_ in the PP model; *K*_in,0_, *K*_out_, and *K*_DA_ in the AAI model) (Mager et al. 2009). For the translational PP model, these scaled parameters were comparable to those of the benchmark PP models (Table 1). Our assumption that RO_50_ does not require translation was based on the pharmacological principle that the drug effect is dependent on RO, irrespective of the compounds, provided that these are full antagonists with a similar mechanism of action. RO_50_ is a single common denominator expressing the receptor blockade required to produce half‐maximal effect, allowing using in vitro KI values to estimate the potency of different compounds. A similar approach was applied to link central D_2_ RO to efficacy and safety (Pilla Reddy 2012). Moreover, the hypothalamo‐pituitary system regulating prolactin release is similar, although the rat dopaminergic system is somewhat more complex (Ben‐Jonathan et al. 2008). Interspecies scaling of model parameters has been applied for the PP model to predict the effect of varying the dosing interval between two successive doses of REM on prolactin response (Stevens et al. 2012). In contrast, our focus was on predicting prolactin response at a clinically relevant dose regimen. Stevens and colleagues applied a sigmoidal function to describe a positive feedback of prolactin on its own synthesis. However, such a function leads to model instability (Bakshi et al. 2016; Taneja et al. 2016a).

In case of the AAI model, the scaled turnover constant for prolactin (*K*_out_) and the baseline plasma levels (*C*_prl_,_0_) were in the human ballpark, but not the scaled turnover constant for dopamine (*K*_DA_) (Table 1). Dopamine levels could not be measured in the preclinical studies, which would have provided a more rational basis for the translation. In published preclinical and clinical studies with the AAI model to date, DAs_0_ has typically been assumed to be 10 000, since higher values resulted in unacceptably long runtimes (Friberg et al. 2009b). We fixed it to a more plausible value of 10.9, estimated from rat data. Since this parameter is a system‐specific parameter and a rationale for interspecies scaling is lacking and the parameters is scaled as it is, we hypothesized that no further scaling was necessary. In the absence of drug, the RO of dopamine (eq. (8)) would be 99.99% if the DAs_0_ were taken as 10 000 but would be 91.6% if it is taken to be 10.9. In other words, in the absence of drug, the concentration of free receptors available for interaction with the drug is extremely low, which seems unlikely from a physiological standpoint.

Petersson and colleagues have shown that in vitro experimental human KI values are highly correlated with corresponding estimates from population data (Petersson et al. 2013). On the other hand, Johnson and colleagues have proposed a scaling which normalizes estimated rat in vivo *K*_d_ to in vitro KI values for rats and humans (Johnson et al. 2016). Hence, we tested both the in vitro KIs as well as the scaled values.

Plasma prolactin predictions for OROS PA with the original Movin‐Osswald PP model (Fig. 7) show differences in peak concentrations with those of the translational PP model owing to different values of the potency parameter used (Fig. 2). What is common, however, is that the pool does not recover sufficiently to output subsequent plasma prolactin peaks following the first dose.

For REM, in contrast, the predictions indicate a drop in RO between doses (Fig. 4). The corresponding plasma prolactin predictions show that following the first dose, complete pool recovery does not occur and subsequent peaks are considerably lower than those at day 1. Predictions with the original Movin‐Osswald model revealed quantitative differences with respect to the translational PP model. The first peak was lower (50 ng·mL^−1^ compared to 120 ng·mL^−1^) and subsequent peaks were higher, indicating quicker recovery of the pool (Fig. 8). In a published PK study with REM, prolactin peak concentrations were in the range of 38–72 ng·mL^−1^ following 50 mg REM (Movin‐Osswald et al. 1995). A second dose after a dosing interval of 24 h results in a second peak almost identical to the first dose. This means that the translational PP model overpredicts prolactin concentration on day 1 and underpredicts thereafter.

For the observation that subsequent peaks occur with REM but not with PA, we found that this is attributable to differences in kinetics, in particular their half‐life. The half‐life of REM is ~5 h which allows at least partial recovery of the pool in‐between doses, whereas for OROS PA the recovery period is longer, as depicted in Figure 3.

Predicted pituitary RO for PA and REM showed closer agreement with published literature as compared to plasma prolactin levels. Predicted pituitary RO for PA compared well with in vivo D_2_ RO based on modeling and simulation of human data (De Ridder 2005). Also, these predictions compared well with the findings of Johnson who predicted human striatal D_2_ RO using a physiology‐based PKPD model and compared predictions with observed data (Fig. 1) (Johnson 2012).

For REM, the predicted pituitary RO was between 80% and 95% for the dose range of 50–600 mg per day, while in PET studies in humans, striatal RO has been reported to be 60–80% (Klemm et al. 1996). Farde and von Bahr reported 73% and 71% striatal RO in human subjects dosed with REM 100 mg thrice daily or 200 mg twice daily, respectively (Farde and von Bahr 1990). For these dosing paradigms, we predicted median RO of 83% (90% prediction interval 74–89%) and 89% (72–93%), respectively.

RO and plasma prolactin concentrations were underpredicted by the translational AAI model for both PA and REM. These underpredictions were in the range of 5–70‐fold for RO and 2–5‐fold for prolactin, indicative of an artifact in the model wherein the predicted RO (eq. (7)) could not explain the predicted prolactin response. If DAs_0_ is 10 000 as has been done in preclinical and clinical publications till now (Friberg et al. 2009b; Ma et al. 2010; Petersson et al. 2012, 2013), D_2_ receptor occupancy by dopamine is nearly 100%, even in the presence of the drug, implying that D2 receptor occupancy by the antipsychotic drug is very low. If this parameter is taken to be 10.9, as estimated from rat data, predictions of RO were higher, but yet remained far below reported occupancy levels. Fixing the DAs_0_ to an arbitrary constant enables description of the data, but limits the predictive ability and translatability of the model. Prolactin predictions are not sensitive to wide variations in the estimate of this parameter (Friberg et al. 2009b). The translational AAI model does not predict the overwhelming tolerance to prolactin response predicted by the translational PP model.

It should be clarified that central (striatal) RO usually reported in literature and the pituitary RO are not always comparable. Kohler and Karlsson‐Boethius (1989) stated that REM is equipotent for blocking receptors in the pituitary and the brain. Kapur et al. (2000) showed that for haloperidol, the narrow therapeutic window of pituitary D_2_ RO cannot be reliably quantified with PET studies, and there was no significant difference between striatal and extrastriatal D_2_ RO. In case of lipophilic antipsychotics, brain concentrations are in rapid equilibrium with plasma concentrations, and they may not undergo active transport during distribution in brain (Matsui‐Sakata et al. 2005). In a PET study to examine RO in humans, it has been shown that 80% of the REM injected peripherally passed through the blood–brain barrier within minutes (Farde and von Bahr 1990). For compounds with a higher blood barrier penetration, pituitary and striatal ROs are comparable or are in a constant ratio. Risperidone (and by extension paliperidone) does not penetrate the blood barrier well due to active efflux by P‐glycoprotein, and hence a higher disassociation between central and neuroendocrine effects can be expected (Kapur et al. 2000).

The significance of predicting plasma prolactin lies in its ability to inform clinical efficacy and extrapyramidal side effects. The RO predicted by the translational PP model is in the same ballpark as that reported in published studies using imaging modalities (Farde and von Bahr 1990; Klemm et al. 1996). Furthermore, a similar approach has been used to predict central RO for PA and RI, respectively (De Ridder 2005; Gomeni et al. 2013). In a study in healthy volunteers dosed with 70 or 140 mg REM intravenously thrice daily for 7 days, seven out of eight subjects on the highest dose reported akathisia on day 7 of the study (Farde et al. 1988).

Our simulations with the translational PP model predict a peak RO of >90% for 200 mg twice daily. It is known that motor side effects appear at RO > 80%, hence the translational PP model would have been able to predict akathisia with this dose regimen.

The incidence of extra‐pyramidal symptoms becomes significantly higher than placebo beyond a dose of 6 mg PA daily (De Ridder 2005). Figure 1 shows that RO at this dose is ~85%, indicative of a conformance between model predictions and published information.

While both models have been published earlier with clinical data, we linked the prolactin time course to receptor occupancy, enabling prediction of efficacy as well as safety. This resulted in the models becoming system‐specific and independent of physico‐chemical properties of the drugs.

In conclusion, while neither model could completely predict prolactin responses in humans, the translational PP model predicted prolactin response after a single dose better than the AAI model. Prolactin pool depletion is a feature of this model, which precludes reliable multiple dose predictions. Pituitary D_2_ RO, however, was reliably predicted and can be the basis for predicting efficacy and motor side effects in humans. The translational AAI model failed to accurately predict RO and plasma prolactin. It is of translational value if an alternative approach is applied, wherein system‐specific parameters are fixed based on published data and KIs derived from in vitro experimental information (Petersson et al. 2013). Based on our findings, we speculate on possible improvements in the modeling approach for future research. In case of the translational PP model, the main drawback is the acute tolerance predicted. Here, alternative mechanisms of tolerance described in literature could be evaluated. In addition, we did not have receptor occupancy data, which would have greatly improved the predictive properties of the model, given that we hypothesized that RO was the driver of the prolactin response, rather than the drug concentrations. In case of the AAI model, measured dopamine concentrations would have provided seminal insights as to the dopamine feedback loop, and possibly led to alternative parametrizations of this pathway. Our effort is a first step toward addressing the complex challenge of interspecies scaling of PD for antipsychotics. Shortcomings of the translations reflect the need for better mechanistic models. For our proposed strategy to be fully applicable to a real‐life situation, it would have to be integrated with physiology‐based pharmacokinetic (PBPK) models (Rostami‐Hodjegan 2012).

## Author Contributions

A. T. participated in the study design, performed the data analysis and interpretation of the results, and wrote the manuscript. A. V. participated in the study design, interpretation of the results, and approved the final manuscript. D. R. H. H. participated in the study design, interpretation of the results, and approved the final manuscript. M. D. participated in the study design, interpretation of the results, and approved the final manuscript. E. C. M. D. participated in the study design, supervised the experimental procedures, participated in the interpretation of the results, and approved the final manuscript. J. H. P. participated in the study design, performed the data analysis and interpretation of the results, and approved the final manuscript.

## Disclosure

None declared.

## Supporting information


**Figure S1.** Precursor pool (PP) model as implemented by Stevens et al. (2012), modified so as to parametrize drug effect (DE) in terms of receptor occupancy (RO).
**Figure S2.** Agonist–antagonist interaction (AAI) model as implemented by Friberg et al. (2009a, b).
**Figure S3.** Predicted RO profiles (left) and plasma prolactin profiles (right) following 100 mg thrice daily (upper panels) or 200 mg twice daily (lower panels) of REM for 8 days in humans with the translational PP model.
**Figure S4.** Predicted typical time course of PA, RI, REM and corresponding observed plasma prolactin concentrations following single IV dosing of RI (2 mg/kg), PA (0.5 mg/kg), REM (4/8/16 mg/kg) or two doses of REM (3.8 mg/kg).
**Figure S5.** Time course of predicted RO_pituitary_ in rats for RI 2 mg/kg (left panels) and REM 3.8 mg/kg (right panels) with the PP model (upper panels) and the AAI model (lower panels), compared to peak RO_pituitary_ (red dots) and central RO (blue dots) reported by Kapur et al. (2002). Note: Kapur et al. used amisulpiride which has similar potency to remoxipride.
**Table S1.** Human PK parameters used for predictions with the PP and AAI models.
**Table S2.** Final parameter estimates for the pool model and interaction model describing the time course of prolactin and the effects of drug thereupon, including the results of a nonparametric bootstrap analysis (*n* = 500). For the pool model, KI values were fixed to the values estimated from the interaction model.

## References

[prp2364-bib-0001] Anderson BJ , Holford NH (2008). Mechanism‐based concepts of size and maturity in pharmacokinetics. Annu Rev Pharmacol Toxicol 48: 303–332. 17914927 10.1146/annurev.pharmtox.48.113006.094708

[prp2364-bib-0002] Bagli M , Suverkrup R , Quadflieg R , Hoflich G , Kasper S , Moller HJ , et al. (1999). Pharmacokinetic‐pharmacodynamic modeling of tolerance to the prolactin‐secreting effect of chlorprothixene after different modes of drug administration. J Pharmacol Exp Ther 291: 547–554. 10525070

[prp2364-bib-0003] Bakshi S , de Lange EC , van der Graaf PH , Danhof M , Peletier LA (2016). Understanding the behavior of systems pharmacology models using mathematical analysis of differential equations: prolactin modeling as a case study. CPT Pharmacometrics Syst Pharmacol 5: 339–351. 27405001 10.1002/psp4.12098 PMC4961077

[prp2364-bib-0004] Beal S , Sheiner LB , Boeckmann A , Bauer RJ (2009). NONMEM's user's guides. ICON Development Solutions, Hanover, MD, USA.

[prp2364-bib-0005] Ben‐Jonathan N , LaPensee CR , LaPensee EW (2008). What can we learn from rodents about prolactin in humans? Endocr Rev 29: 1–41. 18057139 10.1210/er.2007-0017 PMC2244934

[prp2364-bib-0006] Berwaerts J , Cleton A , Rossenu S , Talluri K , Remmerie B , Janssens L , et al. (2010). A comparison of serum prolactin concentrations after administration of paliperidone extended‐release and risperidone tablets in patients with schizophrenia. J Psychopharmacol 24: 1011–1018. 19825908 10.1177/0269881109106914

[prp2364-bib-0007] Boxenbaum H (1982). Interspecies scaling, allometry, physiological time, and the ground plan of pharmacokinetics. J Pharmacokinet Biopharm 10: 201–227. 7120049 10.1007/BF01062336

[prp2364-bib-0008] De Ridder F (2005). Predicting the outcome of phase III trials using phase II data: a case study of clinical trial simulation in late stage drug development. Basic Clin Pharmacol Toxicol 96: 235–241. 15733220 10.1111/j.1742-7843.2005.pto960314.x

[prp2364-bib-0010] Farde L , von Bahr C (1990). Distribution of remoxipride to the human brain and central D2‐dopamine receptor binding examined in vivo by PET. Acta Psychiatr Scand Suppl 358: 67–71. 1978494 10.1111/j.1600-0447.1990.tb05292.x

[prp2364-bib-0011] Farde L , Grind M , Nilsson MI , Ogenstad S , Sedvall G (1988). Remoxipride–a new potential antipsychotic drug. pharmacological effects and pharmacokinetics following repeated oral administration in male volunteers. Psychopharmacology 95: 157–161. 2901121 10.1007/BF00174501

[prp2364-bib-0012] Friberg LE , de Greef R , Kerbusch T , Karlsson MO (2009a). Modeling and simulation of the time course of asenapine exposure response and dropout patterns in acute schizophrenia. Clin Pharmacol Ther 86: 84–91. 19387434 10.1038/clpt.2009.44

[prp2364-bib-0013] Friberg LE , Vermeulen AM , Petersson KJ , Karlsson MO (2009b). An agonist‐antagonist interaction model for prolactin release following risperidone and paliperidone treatment. Clin Pharmacol Ther 85: 409–417. 19109590 10.1038/clpt.2008.234

[prp2364-bib-0014] Gomeni R , Heidbreder C , Fudala PJ , Nasser AF (2013). A model‐based approach to characterize the population pharmacokinetics and the relationship between the pharmacokinetic and safety profiles of RBP‐7000, a new, long‐acting, sustained‐released formulation of risperidone. J Clin Pharmacol 53: 1010–1019. 23868656 10.1002/jcph.141

[prp2364-bib-0015] Johnson M (2012). Translational PKPD modeling in schizophrenia – predicting human receptor occupancy. Doctoral dissertation, University of Groningen, the Netherlands, pp 99–127.

[prp2364-bib-0016] Johnson M , Kozielska M , Pilla Reddy V , Vermeulen A , Barton HA , Grimwood S , et al. (2016). Translational modeling in schizophrenia: predicting human dopamine D2 receptor occupancy. Pharm Res 33: 1003–1017. 26718955 10.1007/s11095-015-1846-4

[prp2364-bib-0017] Kane JM (1993). Newer antipsychotic drugs. A review of their pharmacology and therapeutic potential. Drugs 46: 585–593. 7506647 10.2165/00003495-199346040-00002

[prp2364-bib-0501] Kapur S , Langlois X , Vinken P , Megens AA , De Coster R , Andrews JS (2002). The differential effects of atypical antipsychotics on prolactin elevation are explained by their differential blood‐brain disposition: A pharmacological analysis in rats. J Pharmacol Exp Ther 302: 1129–1134. 12183672 10.1124/jpet.102.035303

[prp2364-bib-0018] Kapur S , Zipursky R , Jones C , Remington G , Houle S (2000). Relationship between dopamine D(2) occupancy, clinical response, and side effects: a double‐blind PET study of first‐episode schizophrenia. Am J Psychiatry 157: 514–520. 10739409 10.1176/appi.ajp.157.4.514

[prp2364-bib-0019] Klemm E , Grunwald F , Kasper S , Menzel C , Broich K , Danos P , et al. (1996). 123I]IBZM SPECT for imaging of striatal D2 dopamine receptors in 56 schizophrenic patients taking various neuroleptics. Am J Psychiatry 153: 183–190. 8561197 10.1176/ajp.153.2.183

[prp2364-bib-0020] Kohler C , Karlsson‐Boethius G (1989). In vivo labelling of pituitary dopamine D‐2 receptors in the male rat using [3H]‐raclopride. J Neural Transm 76: 13–28. 2523467 10.1007/BF01244988

[prp2364-bib-0021] Lepist EI , Jusko WJ (2004). Modeling and allometric scaling of s(+)‐ketoprofen pharmacokinetics and pharmacodynamics: a retrospective analysis. J Vet Pharmacol Ther 27: 211–218. 15305849 10.1111/j.1365-2885.2004.00579.x

[prp2364-bib-0022] Lindbom L , Ribbing J , Jonsson EN (2004). Perl‐speaks‐NONMEM (PsN)–a perl module for NONMEM related programming. Comput Methods Programs Biomed 75: 85–94. 15212851 10.1016/j.cmpb.2003.11.003

[prp2364-bib-0024] Ma G , Friberg LE , Movin‐Osswald G , Karlsson MO (2010). Comparison of the agonist‐antagonist interaction model and the pool model for the effect of remoxipride on prolactin. Br J Clin Pharmacol 70: 815–824. 21175437 10.1111/j.1365-2125.2010.03758.x PMC3014065

[prp2364-bib-0025] Mager D , Jusko WJ (2007) Mechanistic PKPD models II Pp. 607–654 in Ete W., ed. Pharmacometrics. The science of quantitative pharmacology. Wiley Interscience, New Jersey.

[prp2364-bib-0026] Mager DE , Woo S , Jusko WJ (2009). Scaling pharmacodynamics from in vitro and preclinical animal studies to humans. Drug Metab Pharmacokinet 24: 16–24. 19252333 10.2133/dmpk.24.16 PMC3727168

[prp2364-bib-0027] Matsui‐Sakata A , Ohtani H , Sawada Y (2005). Receptor occupancy‐based analysis of the contributions of various receptors to antipsychotics‐induced weight gain and diabetes mellitus. Drug Metab Pharmacokinet 20: 368–378. 16272755 10.2133/dmpk.20.368

[prp2364-bib-0028] Movin‐Osswald G , Hammarlund‐Udenaes M (1995). Prolactin release after remoxipride by an integrated pharmacokinetic‐pharmacodynamic model with intra‐ and interindividual aspects. J Pharmacol Exp Ther 274: 921–927. 7636755

[prp2364-bib-0029] Movin‐Osswald G , Hammarlund‐Udenaes M , Von Bahr C , Eneroth P , Walton‐Bowen K (1995). Influence of the dosing interval on prolactin release after remoxipride. Br J Clin Pharmacol 39: 503–510. 7669486 10.1111/j.1365-2125.1995.tb04487.x PMC1365057

[prp2364-bib-0030] Petersson KV , Ivaturi V , Vermeulen AV , Friberg LE (2012). Simultaneous modeling of prolactin data following administration of seven D2–receptor antagonists in rats; model‐based in vitro – rat – human scaling. Paper presented at the Population Approach Group of Europe 2012, Venice, Italy.

[prp2364-bib-0031] Petersson KJ , Vermeulen AM , Friberg LE (2013). Predictions of in vivo prolactin levels from in vitro K(i) values of D(2) receptor antagonists using an agonist‐antagonist interaction model. AAPS J 15: 533–541. 23392818 10.1208/s12248-012-9450-6 PMC3675734

[prp2364-bib-0032] Peuskens J , Pani L , Detraux J , De Hert M (2014). The effects of novel and newly approved antipsychotics on serum prolactin levels: a comprehensive review. CNS Drugs 28: 421–453. 24677189 10.1007/s40263-014-0157-3 PMC4022988

[prp2364-bib-0033] Pilla Reddy V (2012). Translational PKPD modelling in schizophrenia – linking receptor occupancy of antipsychotics to efficacy and safety. Doctoral dissertation, University of Groningen, the Netherlands, pp 224–252.

[prp2364-bib-0034] R Core Team ed . (2015). R: A language and environment for statistical computing, R Foundation for Statistical Computing, Vienna, Austria. https://www.R-project.org.

[prp2364-bib-0035] Rodriguez‐Martinez A , Quilo CG (2013). Paliperidone extended‐release: safety and tolerability from a metabolic profile perspective. Clin Drug Investig 33: 867–876. 10.1007/s40261-013-0100-2 24241935

[prp2364-bib-0036] Rohatagi A (2014). WebplotDigitizer, version 3.4. http://arohatagi.info/WebPlotDigitizer.

[prp2364-bib-0037] Rostami‐Hodjegan A (2012). Physiologically based pharmacokinetics joined with in vitro‐in vivo extrapolation of ADME: a marriage under the arch of systems pharmacology. Clin Pharmacol Ther 92: 50–61. 22644330 10.1038/clpt.2012.65

[prp2364-bib-0038] Samtani MN , Gopal S , Gassmann‐Mayer C , Alphs L , Palumbo JM (2011). Dosing and switching strategies for paliperidone palmitate: based on population pharmacokinetic modelling and clinical trial data. CNS Drugs 25: 829–845. 21936586 10.2165/11591690-000000000-00000

[prp2364-bib-0039] Stevens J , Ploeger BA , Hammarlund‐Udenaes M , Osswald G , van der Graaf PH , Danhof M , et al. (2012). Mechanism‐based PK‐PD model for the prolactin biological system response following an acute dopamine inhibition challenge: quantitative extrapolation to humans. J Pharmacokinet Pharmacodyn 39: 463–477. 22791078 10.1007/s10928-012-9262-4

[prp2364-bib-0040] Taneja A , Vermeulen A , Huntjens DR , Danhof M , De Lange EC , Proost JH (2016a). A comparison of two semi‐mechanistic models for prolactin release and prediction of receptor occupancy following administration of dopamine D2 receptor antagonists in rats. Eur J Pharmacol 789: 202–214. 27395799 10.1016/j.ejphar.2016.07.005

[prp2364-bib-0041] Taneja A , Vermeulen A , Huntjens DR , Danhof M , De Lange EC , Proost JH (2016b). Summary data of potency and parameter information from semi‐mechanistic PKPD modeling of prolactin release following administration of the dopamine D2 receptor antagonists risperidone, paliperidone and remoxipride in rats. Data Brief 8: 1433–1437. 27617278 10.1016/j.dib.2016.07.060 PMC5007417

[prp2364-bib-0042] Yassen A , Olofsen E , Kan J , Dahan A , Danhof M (2007a). Animal‐to‐human extrapolation of the pharmacokinetic and pharmacodynamic properties of buprenorphine. Clin Pharmacokinet 46: 433–447. 17465641 10.2165/00003088-200746050-00005

[prp2364-bib-0043] Yassen A , Olofsen E , van Dorp E , Sarton E , Teppema L , Danhof M , et al. (2007b). Mechanism‐based pharmacokinetic‐pharmacodynamic modelling of the reversal of buprenorphine‐induced respiratory depression by naloxone: a study in healthy volunteers. Clin Pharmacokinet 46: 965–980. 17922561 10.2165/00003088-200746110-00004

[prp2364-bib-0044] Zuideveld KP , Van der Graaf PH , Peletier LA , Danhof M (2007). Allometric scaling of pharmacodynamic responses: application to 5‐HT1A receptor mediated responses from rat to man. Pharm Res 24: 2031–2039. 17541734 10.1007/s11095-007-9336-y

