# Supplementary material for: Modeling of prolactin response following dopamine D2 receptor antagonists in rats: can it be translated to clinical dosing?
Source: Pharmacol Res Perspect. 2017 Nov 21;5(6):e00364. doi: 10.1002/prp2.364 (PMC5723698; doi:10.1002/prp2.364)
Supplement: Supplementary file 1 — Figure S1. Precursor pool (PP) model as implemented by Stevens et al. (2012), modified so as to parametrize drug effect (DE) in terms of receptor occupancy (RO). Figure S2. Agonist–antagonist interaction (AAI) model as implemented by Friberg et al. (2009a, b). Figure S3. Predicted RO profiles (left) and plasma prolactin profiles (right) following 100 mg thrice daily (upper panels) or 200 mg twice daily (lower panels) of REM for 8 days in humans with the translational PP model. Figure S4. Predicted typical time course of PA, RI, REM and corresponding observed plasma prolactin concentrations following single IV dosing of RI (2 mg/kg), PA (0.5 mg/kg), REM (4/8/16 mg/kg) or two doses of REM (3.8 mg/kg). Figure S5. Time course of predicted ROpituitary in rats for RI 2 mg/kg (left panels) and REM 3.8 mg/kg (right panels) with the PP model (upper panels) and the AAI model (lower panels), compared to peak ROpituitary (red dots) and central RO (blue dots) reported by Kapur et al. (2002). Note: Kapur et al. used amisulpiride which has similar potency to remoxipride. Table S1. Human PK parameters used for predictions with the PP and AAI models. Table S2. Final parameter estimates for the pool model and interaction model describing the time course of prolactin and the effects of drug thereupon, including the results of a nonparametric bootstrap analysis (n = 500). For the pool model, KI values were fixed to the values estimated from the interaction model. [file PRP2-5-e00364-s001.pdf]

Pharmacology Research & Perspectives

Modeling of prolactin response following dopamine D_2 receptor antagonists in rats: Can it be translated to clinical dosing?

A Taneja, A Vermeulen, D R H Huntjens, M Danhof, E C M De Lange, and J H Proost

SUPPLEMENTAL MATERIAL

All tables, figures and equations pertaining to this section are prefixed as S.

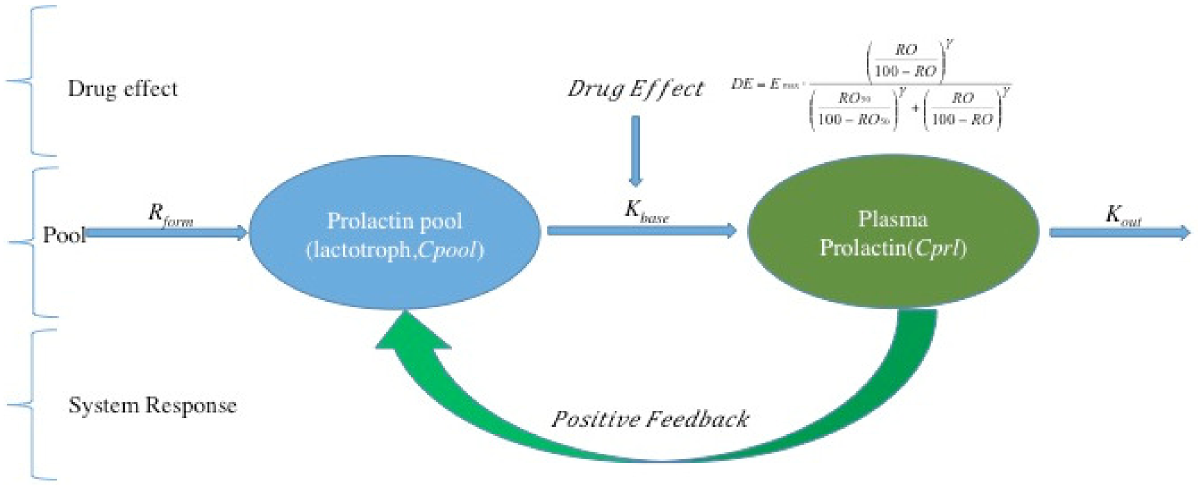

Figure S1. Precursor pool (PP) model as implemented by Stevens et al. (2012), modified so as to parametrise drug effect (DE) in terms of receptor occupancy (RO).

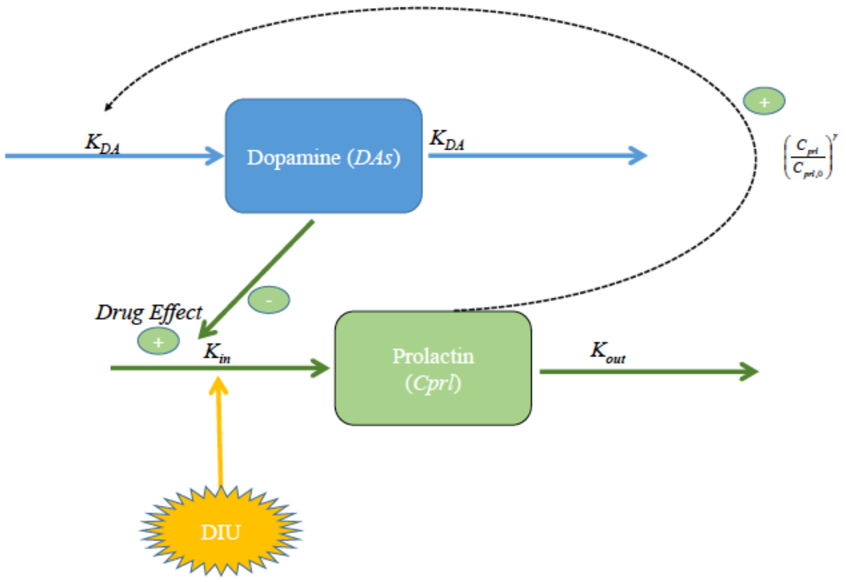

Figure S2. Agonist-antagonist interaction (AAI) model as implemented by Friberg et al. (2009).

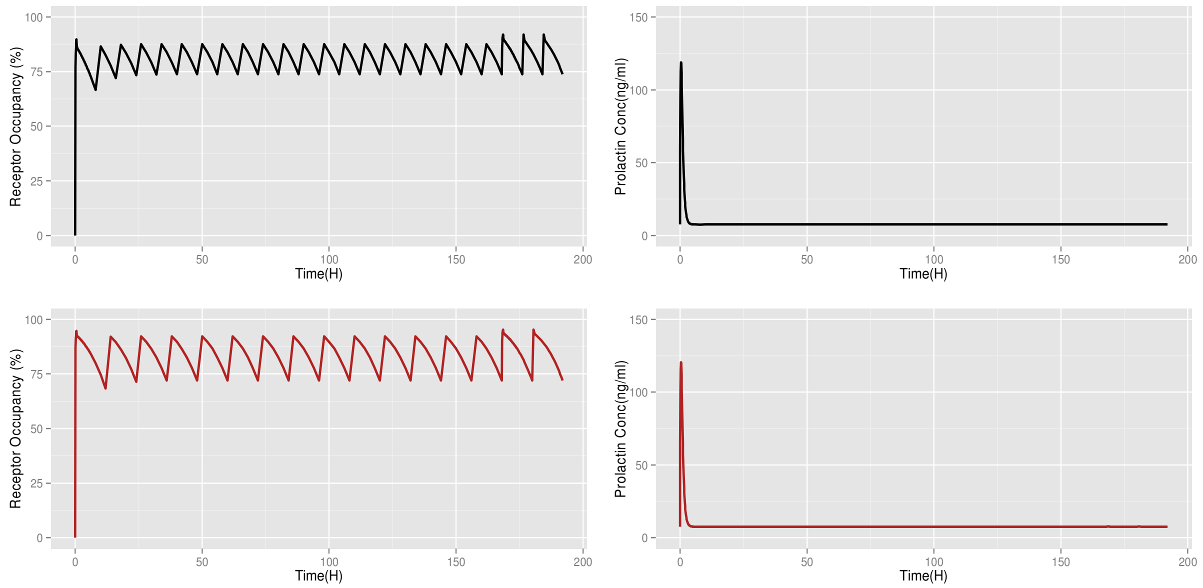

Figure S3. Predicted RO profiles (left) and plasma prolactin profiles (right) following 100 mg thrice daily (upper panels) or 200 mg twice daily (lower panels) of REM for 8 days in humans with the translational PP model.

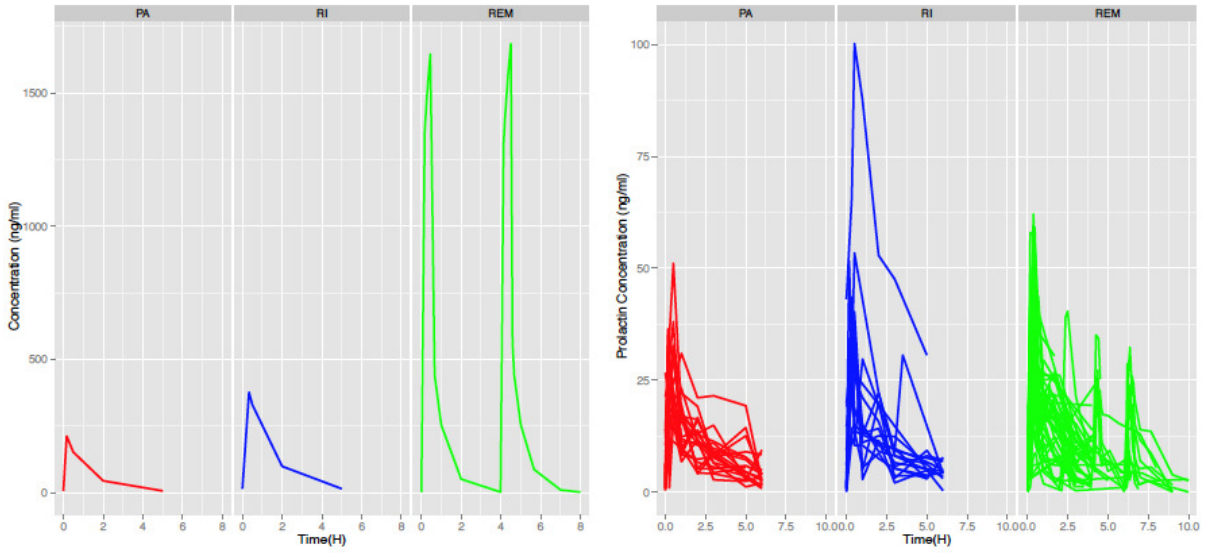

Figure S4. Predicted typical time course of PA, RI, REM and corresponding observed plasma prolactin concentrations following single IV dosing of RI (2 mg/kg), PA (0.5 mg/kg),REM (4/8/16 mg/kg) or two doses of REM (3.8 mg/kg). (*Adapted with permission from Taneja et al., European Journal of Pharmacology 2016; 789: 202-214*).

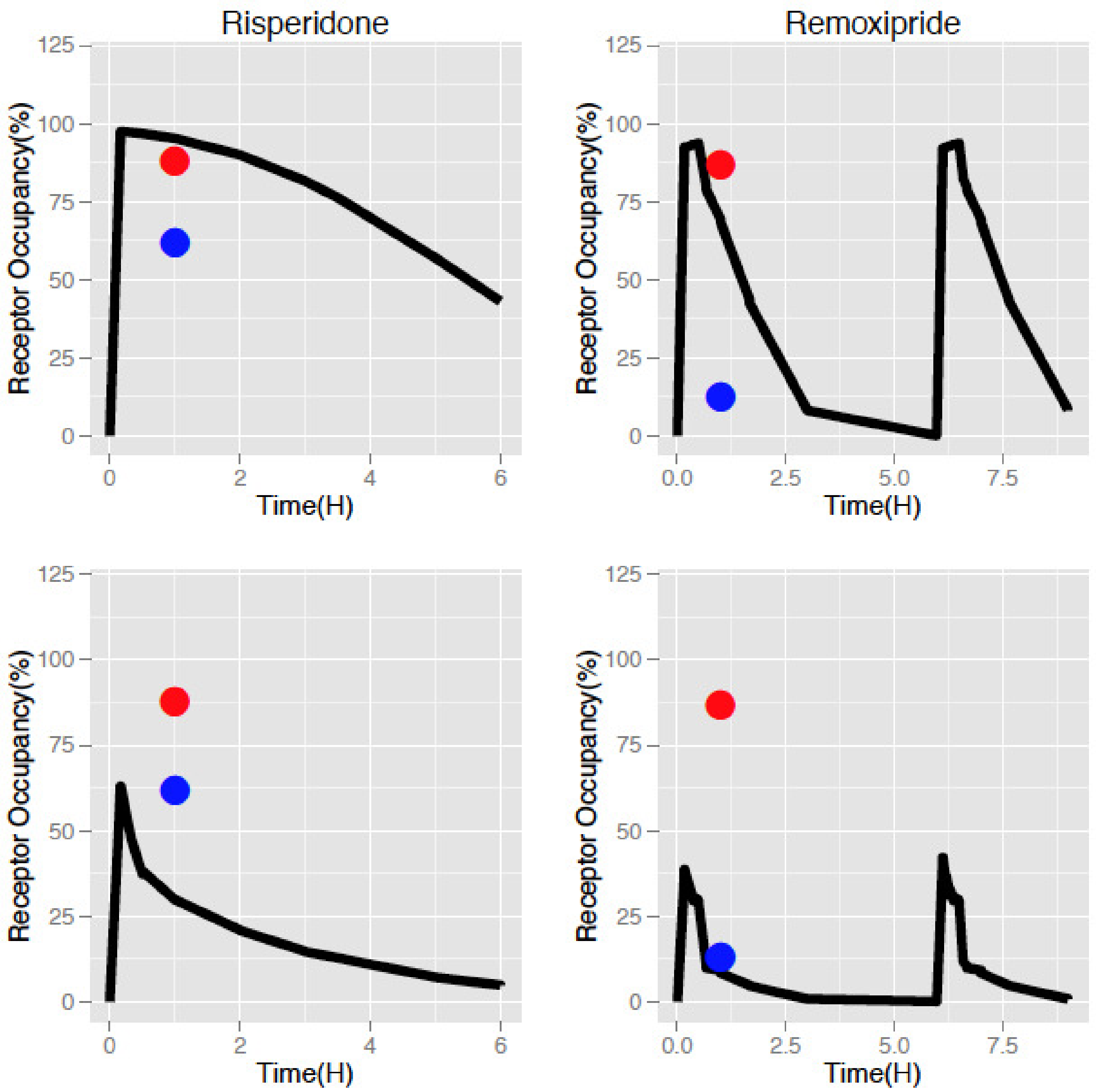

Figure S5. Time course of predicted $RO_{pituitary}$ in rats for RI 2 mg/kg (left panels) and REM 3.8 mg/kg (right panels) with the PP model (upper panels) and the AAI model (lower panels), compared to peak $RO_{pituitary}$ (red dots) and central RO (blue dots) reported by Kapur et al. (2002). Note: Kapur et al. used amisulpiride which has similar potency to remoxipride. (*Adapted with permission from Taneja et al., European Journal of Pharmacology 2016; 789: 202-214*).

Table S1. Human PK parameters used for predictions with the PP and AAI models.

**Parameter**	**Compound**	
	**Paliperidone**	**Remoxipride**
CL ($l.h^{-1}$)	14.15	7.44
V_1 (l)	395.4	122
Q ($l.h^{-1}$)		137
V_2 (l)		34.7
Tdur (h)	22.87	
Ka (h^{-1})	2.49	
Molecular weight	426.48	371.26

V_1 = volume of the central compartment; V_2 = volume of the peripheral compartment; *CL* = clearance from the central compartment; *Q* = inter-compartmental clearance; *Tdur* = duration of zero-order release; *Ka* = first-order absorption rate constant.

Table S2. Final parameter estimates for the pool model and interaction model describing the time course of prolactin and the effects of drug thereupon, including the results of a nonparametric bootstrap analysis (n=500). For the pool model, *KI* values were fixed to the values estimated from the interaction model. (*Adapted with permission from Taneja et al., European Journal of Pharmacology 2016; 789: 202-214*).

				Bootstrap Results		
**Pool Model**		**RSE%**	**Median**	**Lower Limit 95% CI**	**Upper Limit 95% CI**
R_{form} (ng.ml^{-1}.h^{-1})	49.3	10	50.8	42.0	62.0
K_{base} (h^{-1})	0.237	11	0.230	0.178	0.301
K_{out} (h^{-1})	6.64	13	6.98	5.38	8.70
E_{max}	3.5 FIXED				
RO_{50} (%)	56.3	14	56.0	37.4	71.9
KI risperidone/ paliperidone (nM)	11.1 FIXED				
KI remoxipride (nM)	113 FIXED				
IIV K_{out} (%)	42.0	18	41.6	33.4	49.2
Proportional error (%)	42.2	7	38.6	29.9	44.6
Additive error (ng.ml^{-1})	2.42	30	2.37	1.52	3.66
**Interaction Model**					
$K_{in,0}$ (ng.ml^{-1}.h^{-1})	30.2	22	32.4	16.1	84.5
K_{out} (h^{-1})	5.78	20	6.61	3.72	13.5
K_{DA} (h^{-1})	3.92	26	3.50	0.19	5.38
DAs_0	10.9	41	9.38	4.34	81.2
KI risperidone/ paliperidone (nM)	11.1	21	11.1	5.58	31.3
KI remoxipride (nM)	113	27	114	18.7	264
IIV K_{out} (%)	41.0	9	40.5	33.0	48.4
Proportional error (%)	29.3	9	28.8	23.7	33.7
Additive error (ng.ml^{-1})	4.65	11	4.52	4.00	5.14

Parametrisation of the relationship between pituitary receptor occupancy and drug effect

Once the final PP model had been developed, this model was modified to relate drug effect to pituitary receptor occupancy (*RO*), rather than to unbound drug concentration. The general expression which relates *RO* to drug concentration is (Matsui-Sakata et al., 2005):

$$RO = \frac{Cu}{KI + Cu} \cdot 100 \qquad \text{(S1)}$$

where *RO* is the receptor occupancy (in %), *Cu* is the unbound drug concentration and *KI* is the equilibrium dissociation constant of a ligand determined in inhibition studies. From eq. (S1) it follows upon rearrangement:

$$Cu = KI \cdot \frac{RO}{100 - RO} \qquad \text{(S2)}$$

If RO_{50} is defined as *RO* for *Cu* = ECu_{50} it follows that:

$$ECu_{50} = KI \cdot \frac{RO_{50}}{100 - RO_{50}} \qquad \text{(S3)}$$

Substituting eqs. (S2) and (S3) in eq. (3) results in:

$$DE = E_{\max} \cdot \frac{\left(\frac{RO}{100 - RO}\right)^{\gamma}}{\left(\frac{RO_{50}}{100 - RO_{50}}\right)^{\gamma} + \left(\frac{RO}{100 - RO}\right)^{\gamma}} \qquad \text{(S4)}$$

For drugs with a similar mechanism of action, it may be assumed that the relationship between drug effect and *RO* is independent of the compound. Consequently, RO_{50} is a compound-independent system-specific parameter.

Derivation of receptor occupancy for the drug as well as for dopamine, with the AAI model

KI refers to the equilibrium dissociation constant of a ligand determined in inhibition studies. As per receptor theory, at equilibrium:

$$KI = \frac{Cu \cdot [free\ receptors]}{[drug - bound\ receptors]} \tag{S5}$$

where *Cu* is the unbound drug concentration and [*receptors*] is the receptor density. Thus

$$\frac{Cu}{KI} = \frac{[drug - bound\ receptors]}{[free\ receptors]} \tag{S6}$$

The same holds for dopamine, as agonist (symbol *Kd*):

$$Kd = \frac{Cu_{\text{dopamine}} \cdot [free\ receptors]}{[dopamine - bound\ receptors]} \tag{S7}$$

We define *DAs* as:

$$DAs = \frac{Cu_{\text{dopamine}}}{Kd} \tag{S8}$$

It follows that:

$$DAs = \frac{[dopamine - bound\ receptors]}{[free\ receptors]} \tag{S9}$$

RO is defined as the receptor occupancy of the D_2 antagonist, in %:

$$RO = \frac{[drug - bound\ receptors]}{[total\ receptors]} \cdot 100 \tag{S10}$$

RO_{dopamine} is defined as the receptor occupancy of dopamine, in %:

$$RO_{\text{dopamine}} = \frac{[dopamine - bound\ receptors]}{[total\ receptors]} \cdot 100 \tag{S11}$$

The fraction of free receptors, in %, is:

$$fraction\ free\ receptors = \frac{[free\ receptors]}{[total\ receptors]} \cdot 100 \tag{S12}$$

where

$$[total] = [dopamine - bound] + [drug - bound] + [free] \tag{S13}$$

From eqs. (S6) and (S10) it follows:

$$RO = \frac{\frac{Cu}{KI} \cdot [free\ receptors]}{[total\ receptors]} \cdot 100 \tag{S14}$$

Rearranging Eq. (S13) by dividing both sides by [free]:

$$\frac{[total]}{[free]} = \frac{[dopamine - bound]}{[free]} + \frac{[drug - bound]}{[free]} + \frac{[free]}{[free]} \tag{S15}$$

Replacing terms in eqs. (S6) and (S9) in eq. (S15) leads to:

$$\frac{[total]}{[free]} = DAs + \frac{Cu}{KI} + 1 \tag{S16}$$

From eqs. (S14) and (S16) it follows:

$$RO = \frac{\frac{Cu}{KI}}{DAs + \frac{Cu}{KI} + 1} \cdot 100 \tag{S17}$$

Similarly for the *RO* of dopamine:

$$RO_{\text{dopamine}} = \frac{DAs}{DAs + \frac{Cu}{KI} + 1} \cdot 100 \tag{S18}$$

and the fraction of free receptors:

$$\textit{fraction free receptors} = \frac{1}{DAs + \frac{Cu}{KI} + 1} \cdot 100 \tag{S19}$$

Friberg and colleagues have defined *DAs* as the hypothetical dopamine concentration scaled as multiples of the dopamine concentration inhibiting 50% of the prolactin release (Friberg et al., 2009). This is the same as the definition in eq. (S8), when 50% receptor inhibition occurs for RO_{dopamine} = 50%.

The relationship between drug concentration and effect follows from the following expression (Friberg et al., 2009):

$$K_{in} = K_{in,\, max} \cdot \left(1 - \frac{DAs}{DAs + \frac{Cu}{KI} + 1}\right) + C_{prl,0} \cdot K_{out} \cdot f(DIU) \tag{S20}$$

which in the absence of a diurnal rhythm (DIU) can be written using eq. (S18):

$$K_{in} = K_{\text{in, max}} \cdot \left(1 - RO_{\text{dopamine}}\right) \tag{S21}$$

References

Friberg LE, Vermeulen AM, Petersson KJ, and Karlsson MO (2009) An agonist-antagonist interaction model for prolactin release following risperidone and paliperidone treatment. *Clin Pharmacol Ther* **85**: 409-417, 10.1038/clpt.2008.234 [doi].

Kapur S, Langlois X, Vinken P, Megens AA, De Coster R, and Andrews JS (2002) The differential effects of atypical antipsychotics on prolactin elevation are explained by their differential blood-brain disposition: A pharmacological analysis in rats. *J Pharmacol Exp Ther* **302**: 1129-1134, 10.1124/jpet.102.035303 [doi].

Matsui-Sakata A, Ohtani H, and Sawada Y (2005) Receptor occupancy-based analysis of the contributions of various receptors to antipsychotics-induced weight gain and diabetes mellitus. *Drug Metab Pharmacokinet* **20**: 368-378, JST.JSTAGE/dmpk/20.368 [pii].

Stevens J, Ploeger BA, Hammarlund-Udenaes M, Osswald G, van der Graaf PH, Danhof M, and de Lange EC (2012) Mechanism-based PK-PD model for the prolactin biological system response following an acute dopamine inhibition challenge: quantitative extrapolation to humans. *J Pharmacokinet Pharmacodyn* **39**: 463-477, 10.1007/s10928-012-9262-4 [doi].

Taneja A, Vermeulen A, Huntjens DR, Danhof M, De Lange EC, and Proost JH (2016) A comparison of two semi-mechanistic models for prolactin release and prediction of receptor occupancy following administration of dopamine D2 receptor antagonists in rats. *Eur J Pharmacol* **789**: 202-214, 10.1016/j.ejphar.2016.07.005 [doi].
